# Breastfeeding and weaning practices among mothers in Ghana: A population-based cross-sectional study

**DOI:** 10.1371/journal.pone.0259442

**Published:** 2021-11-12

**Authors:** Prince Kubi Appiah, Hubert Amu, Eric Osei, Kennedy Diema Konlan, Iddris Hadiru Mumuni, Orish Ndudiri Verner, Raymond Saa-Eru Maalman, Eunji Kim, Siwoo Kim, Mohammed Bukari, Hajun Jung, Philip Kofie, Martin Amogre Ayanore, Gregory Kofi Amenuvegbe, Martin Adjuik, Elvis Enowbeyang Tarkang, Robert Kaba Alhassan, Ernestina Safoa Donkor, Francis Bruno Zotor, Margaret Kweku, Paul Amuna, John Owusu Gyapong, So Yoon Kim

**Affiliations:** 1 School of Public Health, University of Health and Allied Sciences, Hohoe, Ghana; 2 Department of Medical Law and Ethics, Asian Institute for Bioethics and Health Law, College of Medicine, Yonsei University, Seoul, Republic of Korea; 3 Department of Public Health, Graduate School of Public Health, Yonsei University, Seoul, Republic of Korea; 4 School of Nursing and Midwifery, University of Health and Allied Sciences, Ho, Ghana; 5 College of Nursing, Yonsei Graduate School, Yonsei University, Seoul, Republic of Korea; 6 School of Medicine, University of Health and Allied Sciences, Ho, Ghana; 7 Korea Foundation for International Healthcare Ghana Office, Accra, Ghana; 8 Yonsei University - University of Health and Allied Sciences Partnership Project Office, Ho, Ghana; 9 Directorate of International Affairs, University of Health and Allied Sciences, Ho, Ghana; 10 Office of the Vice-Chancellor, University of Health and Allied Sciences, Ho, Ghana; Universidade de Sao Paulo Faculdade de Saude Publica, BRAZIL

## Abstract

**Background:**

Children need good nutrition to develop proper immune mechanisms and psychosocial maturity, but malnutrition can affect their ability to realize this. Apart from the national demographic and health survey, which is carried out every 5 years, there have not been enough documented studies on child breastfeeding and weaning practices of caregivers in the Volta Region. We, therefore, examined child breastfeeding and weaning practices of mothers in the Volta Region of Ghana.

**Methods:**

A sub-national survey method was adopted and a semi-structured questionnaire was used to collect data from 396 mothers and their children. Descriptive and inferential statistics comprising frequency, percentage, chi-square, and logistic regression were employed in analysing the data. We defined exclusive breastfeeding as given only breast milk to an infant from a mother or a wet nurse for six months of life except drops or syrups consisting of vitamins, minerals, supplements, or medicines on medical advice, and prolonged breastfeeding as breastfeeding up to 24 months of age.

**Results:**

The prevalence of exclusive breastfeeding (EBF) was 43.7%. Mothers constituting 61.1% started breastfeeding within an hour of giving birth. In addition to breast milk, 5.1% gave fluids to their children on the first day of birth. About 66.4% started complementary feeding at 6 months, 22.0% breastfed for 24 months or beyond, while 40.4% fed their children on-demand. Child’s age (AOR: 0.23, 95% CI:0.12–0.43, p<0.0001), prolonged breastfeeding (AOR: 0.41, 95%CI: 0.12–0.87, p = 0.001), mother’s religion (AOR: 3.92, 95%CI: 1.23–12.61, p = 0.021), feeding practices counselled on (AOR: 1.72, 95%CI: 1.96–3.09, p = 0.023), mother ever heard about EBF (AOR: 0.43, 95%CI: 1.45–2.41, *p* = 0.039), child being fed from the bottle with a nipple (AOR: 1.53, 95%CI: 1.94–2.48, *p* = 0.003), and age at which complementary feeding was started (AOR: 17.43, 95%CI: 3.47–87.55, *p* = 0.008) were statistically associated with EBF.

**Conclusion:**

Breastfeeding education has been ongoing for decades, yet there are still gaps in the breastfeeding practices of mothers. To accelerate progress towards attainment of the sustainable development goal 3 of ensuring healthy lives and promoting well-being for all at all ages by the year 2030, we recommend innovative policies that include extensive public education to improve upon the breastfeeding and weaning practices of mothers.

## 1. Background

Globally, progress has been made over the past two decades to improve the nutritional status of all people, including children. However, malnutrition among children continues to be a public health challenge that threatens achievement of the Sustainable Development Goal 3, which is to ensure healthy lives and promote the well-being of all ages by the year 2030 [1]. Globally, about 22.2%, 7.5%, and 5.6% of children are stunted, wasted, and overweight respectively, inclusive of Ghana which has 18.8% stunted, 4.7% wasted, and 2.6% overweighed children [1, 2]. The consequences of malnutrition if not effectively addressed can have dire negative effects on the children including amplified risk of grave ailment and death, often resulting from infectious diseases [3]. This is mainly due to the impact that nutrient deficiencies have on the hematopoietic and lymphoid organs which compromise the inborn and adaptive immune functions [4, 5]. The effects of malnutrition not only affect children but the households, communities, and nations as well [1].

Although, the world is faced with a high prevalence of childhood malnutrition and associated health, economic, and social consequences. There are cost-effective interventions including but not limited to exclusive breastfeeding (EBF), appropriate complementary feeding practices, and prolonged breastfeeding for twenty-four months to overcome the challenge of malnutrition [6–10]. Though these interventions are easy to execute, available data have shown that globally, 42.4% of infants started breastfeeding within an hour of birth, while 40.7% exclusively breastfed for six months. Also, 68.5% started complementary feeding at the sixth month of birth, 45.1% breastfed for two years, and 25.4% had a diversity of five or more food groups [1]. In Ghana, previous studies have shown that 56% of infants were initiated to breastfeeding within an hour after birth and 52% exclusively breastfed. Again, 73% started complementary feeding at the sixth month, 14% bottle-feed, and 50% breastfeed for twenty-four months [11, 12]. In sub-Sahara Africa, the overall prevalence of exclusive breastfeeding is 36.0%, with the highest and lowest prevalence reported in Rwanda and Gabon, respectively [13].

Many factors are influencing the early initiation of breastfeeding and exclusive breastfeeding for six months in Africa. A study in Ethiopia indicated that rural residence, no antenatal follow up, caesarean birth, and home delivery were factors associated with the late start of breastfeeding [14]. Globally, studies have shown that demographics including rural residence, ethnicity, type of employment, richer household wealth quantile and religion, 4+ antenatal care visits, maternal education, knowledge on exclusive breastfeeding, socio-cultural, socioeconomic, birth in a health facility, social support and psychosocial support influence exclusive breastfeeding [13, 15–18]. In developing countries, maternal employment, perceptions of inadequate breast milk supply, mother or infant illnesses, and breast problems are identified as factors that prevent exclusive breastfeeding. Also, socio-cultural factors including, maternal and significant other’s beliefs about infant nutrition, always create barriers to exclusive breastfeeding [19].

Focused antenatal care (FANC) is timely and friendly personalized safe services and care given to a pregnant woman. It focuses on the women’s overall health status, including preparation for childbirth and readiness for complications. Due to the limited number of midwives in Ghana, Community Health Nurses (CHN) and Enrolled Nurses (Auxiliary Nurses) are usually the health workforce during antenatal care visits. Though there is a shortage of midwives, some interventions have been implemented in Ghana to ensure that children are breastfed exclusively for six months. These interventions include mother-to-mother support groups (MMSGs), baby-friendly hospital initiative (BFHI), the Kangaroo mother care programme, and community-based health planning and services (CHIPS) programme [20]. Although prospects are there during the antenatal visits, counselling mothers on breastfeeding is not done regularly [20], notwithstanding the recommendations that the prime emphasis of antenatal care intercessions should be on improving mother and infant health [21]. A study in central Ghana indicated that almost half of pregnant women did not receive information on breastfeeding and concluded that the situation is likely to affect the promotion and breastfeeding support [22]. Investigating knowledge levels of health professionals, Nsiah-Asamoah [23] reported that counselling mothers on appropriate child feeding practices were a problem for these health professionals. Several obstacles affecting the effective delivery of nutritional care in the antenatal and postnatal care settings by health workers have been identified. These include inadequate confidence in nutrition care, insufficient nutrition training during school, poor nutrition-related knowledge, and lack of resources and time [24–27].

Children need good nutrition to develop proper immune mechanisms and psychosocial maturity, but malnutrition can affect their ability to realize this. However, apart from the national demographic and health survey, which is carried out every 5 years [12], there is a paucity of empirical studies on child breastfeeding and weaning practices of caregivers in the Volta Region. Inspired by the pressing need to bridge the data gap, the study assessed breastfeeding and weaning practices of mothers in the Volta Region of Ghana.

## 2. Methods and materials

### 2.1 Study site

The study was carried out in the Volta Region, which is one of the sixteen administrative regions in Ghana. The region is located along the south-eastern part of the country, and shares boundaries with the Republic of Togo to the east, to the west with Greater Accra and Eastern regions, to the north with the Oti Region, and with the Gulf of Guinea to the south. The region has a total land area of about 20,570km^2^; about 8.7% of the total land area of the country [12]. The Volta Region has seventeen administrative districts with a total of five hundred and twenty-six health institutions (GHS Annual Report, 2019 [Unpublished]), serving an estimated population of 1,865,332, with children under five years estimated to be 261,147 (14.0%) [28]. The region is eighth populated region in Ghana [28] and the fourth-lowest region, with 4 per cent of the population in the highest wealth quintile after the Upper West (3.0%), Northern (2.0%), and Upper East (1.7%) regions in descending order. These figures are lower than the national average of the highest wealth quintile (20.0%) [12]. The region has a 64.9% female literacy rate, sixth in the country but lower than the national average of 67.1% [12]. About 71.2% of people in Ghana are Christians, followed by Islam (17.6%) and Traditionalists (5.2%). Christianity dominates the southern part of Ghana, where the Volta region is, and the northern zone is more of Islam [29].

### 2.2 Study population

The study population comprised of mothers who have children between 0 to 59 months and lived in the selected districts. With this, all mothers who were met in the selected households, who agreed to be part of the study, and signed the informed consent forms were recruited. Severely sick people were excluded from the study. Three hundred and ninety-six (396) mothers with children less than five years were eligible for the study, and all participated in the study.

### 2.3 Study design

This study was a community-based descriptive cross-sectional assessment of child feeding practices and factors influencing the conditions of the practice among mothers in three districts (Hohoe, Ho West, and Ketu South) in the Volta Region, and adopted a population-based data gathering to employed participants.

### 2.4 Sampling and sample size determination

A sample size of 396 mothers and their children was determined based on 1,306 households and children less than five years in the selected households in the three administrative districts (Hohoe, Ketu South, and Ho West) randomly selected in the region. According to the 2010 Population and Health Census of Ghana, the total number of households in Hohoe, Ketu South, and Ho West were 43,329, 39,119, and 23,875 respectively. Yamane’s (1998) formulae for population-based sample size determination was then applied [30], to determine the number of households for the districts as indicated below:

Formula: $n=\frac{N}{1+{N(e)}^{2}}$. Where n is defined as the sample size to be determined, N is study population, and e is the level of precision (0.05) at 95% confidence level. Using a 10% non-response rate, the number of households required for each district were 436, 433, and 437 for Ketu South, Ho West, and Hohoe, respectively.

A multi-stage cluster sampling method was applied to select the households for the study. The seventeen districts in the Volta region were categorized into three ecological zones (Northern, Middle, and Coastal zones). A simple random sampling procedure was then employed to pick a district each from the Northern (Hohoe), Middle (Ho West), and the Coastal (Ketu South) zones. In each district, thirty communities were randomly selected, and proportionate allocation was applied to allocate the number of households to each community. The data collectors picked the first housing unit by moving clockwise from the centre of the community. After the first house, the team followed a specific direction to select the next housing unit. Upon entering a housing unit, the first household met was recruited. Where there was no child under five years in the first household, the next household was entered until a targeted child was found in the housing unit. Where there were no targeted children in a housing unit, the next housing unit was entered and the same procedure followed to identify a targeted child for the study.

### 2.5 Data collection tools and procedure

A pre-tested semi-structured questionnaire was used to collect data from respondents. Data collectors were trained and the questionnaire used to collect data from caregivers with children 0–59 months on caregiver education, occupation, education on child care, breastfeeding and infant and young child feeding practices, and childcare practices. The research instrument was interviewer-administered using Computer Assisted Personal Interviews (CAPI) called “RedCap” installed on data collectors’ smartphones and executed through face-to-face interview technique in the homes of the participants. Data were collected on explanatory variables which comprised district, household size, age of both mother and child, mother’s tribe, religion, educational status, occupation, healthcare services mother received towards breastfeeding and care including whether the mother received education on childcare and breastfeeding during the post-natal visit, and whether mother was counselled on child feeding practices. We also collected data on the following variables: Time mothers started breastfeeding after giving birth. In addition to breast milk, what else mother gave to the child on the first day after giving birth. Whether mother have ever heard about EBF. How many times mother breastfed the child in a day. Whether the child ever fed from the bottle with a nipple. The appropriate age of a child when mother think can start complementary feeding, and months the child should breastfed before weaned off from breast. Our outcome variable of interest was EBF for six months. Probing and follow-up questions were used to limit recall bias. The study was between 13^th^ October 2018 and 8^th^ February 2019.

### 2.6 Data analysis

Data collected were entered and cleaned with Statistical Package for Social Sciences (SPSS) V 27 and transported into STATA 14 version for analysis. Descriptive statistics (frequency, percentage) were used in analysing data and presented in frequency tables, cross-tabulations, and charts. Pearson’s chi-square and binary logistic regression models were employed to assess associations between explanatory and outcome variables (EBF). We used a 5% significance level for statistical analysis. Exclusive breastfeeding (EBF) was classified as apart from medicine prescribed by qualified health personnel mother has given only breast milk to the child from birth till six months after birth, and prolonged breastfeeding as breastfeeding up to 24 months of age.

### 2.7 Ethical issues

Ethical clearance for the study was provided by the Research Ethics Committee of the University of Health and Allied Sciences (UHAS) in Ghana (ID: UHAS-REC A.6[7] 17/18). Permission was obtained from the District Health Directorates and Community Leaders (including Chiefs and Assembly members) to carry out the study in their jurisdictions. Participant consent was obtained through the signing/thumb-printing of an informed consent form.

## 3. Results

### 3.1 Background information of participants

Three hundred and ninety-six (396) caregivers and their children under 5 years of age were involved in the study. The median age of the mothers was 29.0 years (IQR: 34.0–23.3), and that of the children was 18 months (IQR: 36.0–7.0). The comparative majority (44.7%) of the caregivers were between the ages of 20–29 years, while the children were 25–59 months (39.1%). A comparative majority of the participants (37.6%) were from the Ketu-South district, followed by Ho-West (32.8%) and Hohoe (29.6%) districts; households with 1–5 members formed the majority (60.9%) in the study. More than half (54.8%) of the children were males; five ethnic groups were involved in the study with the majority (91.9%) being Ewes. Most of the respondents were Christians (91.9%) and had attained basic education (72.2%). Mothers constituting 45.7% were involved in petty trading as their main occupation (Table 1).

**Table 1:** Background information of participants.

Variables	Frequency	Percentage
**District**		
Ketu South	149	37.6
Hohoe	117	29.6
Ho West	130	32.8
**Household size**		
1–5	241	60.9
6–10	134	33.8
11–15	21	5.3
**Age of mothers (years)**		
<20	43	10.9
20–29	177	44.7
30–39	156	39.4
≥40	20	4.1
**Sex of child**		
Male	217	54.8
Female	179	45.2
**Age group of children (months)**		
0–6	97	24.5
7–12	60	15.2
13–24	84	21.2
25–59	155	39.1
**Ethnicity**		
Ewe	364	91.9
Akan	7	1.8
Guan	12	3.0
Kokomba	6	1.5
Manprussi	7	1.8
**Religion**		
Christianity	364	91.9
Islam	14	3.5
Traditionalist	18	4.5
**Educational Status**		
None	25	6.3
Basic	286	72.2
Secondary	63	15.9
Tertiary	22	5.6
**Occupation**		
Artisan	85	21.5
Farmer	45	11.4
Government Employee	22	5.6
Petty trader	181	45.7
Unemployed	63	15.9

### 3.2 Healthcare services mothers received towards breastfeeding and child care

Comparative majority of the mothers (41.9%) received ante-natal care (ANC) during their last pregnancy from a public health centre. Also, 69.9% attended the first ANC facility in the first three months of pregnancy. Although all the mothers went for ANC before given birth, 85.1% of them attended four or more times, whilst 3.5% did so once. About six in ten (58.8%) received advice on baby care during the ANC visits. Of 63.4% who were counselled on EBF, 9.8% did not remember the type of feeding practices they were advised on. After child birth, 45.2% made four or more post-natal care (PNC) visits, 33.3% made one visit, and 1.8% did not visit at all. Of those who went for post-natal care, 27.8% were advised on baby care (Table 2).

**Table 2:** Healthcare services mothers received towards breastfeeding and child care.

Variable	Frequency	Percentage
**Health facility attended for ANC**		
CHPS Centre	21	5.3
Health Centre	166	41.9
District Hospital	153	38.6
Regional Hospital	25	6.3
Private Clinic/Hospital	31	7.8
**Gestational age at first ANC visit (months)**		
1–3	277	69.9
4–6	73	18.4
7–9	7	1.8
Don’t know	39	9.8
**Number of ANC visits before giving birth**		
One time	14	3.5
Two times	31	7.8
Three times	14	3.5
Four or more times	337	85.1
**Received advise on baby care during ANC visit**		
Yes	233	58.8
No	163	41.2
**Feeding practice advised on by the nurse**		
Mixed feeding	95	24.0
Complementary feeding	11	2.8
EBF	251	63.4
Don’t remember	39	9.8
**Number of PNC visits**		
Once	132	33.3
Twice	53	13.4
Thrice	25	6.3
Four or more	179	45.2
None	7	1.8
**Counselled on baby care during PNC visits (n = 389)**		
Yes	108	27.8
No	287	72.2

### 3.3 Breastfeeding and weaning practices of mothers involved in the study

Most of the mothers (61.1%) started breastfeeding within an hour after birth. Also, 5.1% of the women gave fluids apart from breast milk to their babies on the first day of birth. About 11% of the mothers said they have never heard of EBF, while 43.7% noted they had ever or were currently practising EBF. Also, 40.4% breastfed on-demand in a day, 25.3% feed nine or more times, 19.4% feed five to eight times, and 14.9% feed one to four times per day. Almost half of the mothers (47.7%) ever fed their babies from a bottle with a nipple, while 66.4% think appropriate age to start complementary feeding is 6 months. About 56.8% of the mothers used a feeding bottle as part of utensils used to feed their children. Of the children 0–24 months old, 30.3% had been weaned off breastfeeding, with 22.0% of all mothers prolonging breastfeeding for 24 months or beyond before weaning. About 48.5% and 63.9% of the children have completed vitamin A supplementation and immunization respectively required for their age (Table 3).

**Table 3:** Breastfeeding and weaning practices of mothers involved in the study.

Variables		Frequency	Percentage
**Time breastfeeding started after birth**	< 1 hour	242	61.1
	1–5 hours	106	26.8
	6–24 hours	16	4.0
	After a day	32	8.1
**What was given to baby in the first day of birth**	Other fluids	20	5.1
	Breast milk	376	94.9
**Caregiver ever heard about EBF**	Yes	352	88.9
	No	44	11.1
**Currently/ever practice EBF**	Yes	173	43.7
	No	223	56.3
**Number of times breastfeed/feed infant in a day**	1–4	59	14.9
	5–8	77	19.4
	More than 8	100	25.3
	On demand	160	40.4
**Child ever drunk anything from a bottle with a nipple**	Yes	189	47.7
	No	207	52.3
**Appropriate age to start complementary feeding**	< 1 month	24	6.1
	1–3 months	21	5.3
	4–5 months	76	19.2
	At 6 months	263	66.4
	Don’t know	12	3.0
**Utensils used to give complementary food to infants**	Feeding bottle	225	56.8
	Plate	218	55.1
	Spoon	127	32.1
	Cup	115	29.0
**Prolonged breastfeeding (n = 241)**	Yes	168	69.7
	No	73	30.3
**Duration for breast feeding a child**	1–6 months	84	21.2
	7–12 months	31	7.8
	13–18 month	54	13.6
	19–23 months	121	30.6
	24 or more months	87	22.0
	Don’t know	19	4.8
**Child complete Vitamin A supplementation at age**	Yes	192	48.5
	No	186	51.5
**Child complete Immunization at age**	Yes	253	63.9
	No	130	46.1

### 3.4 Associations between background characteristics and EBF

The bivariate analysis showed significant associations between EBF and age of child (*p* < 0.001) and religion (*p* = 0.022). Besides, when the variables were adjusted for confounding effects using multivariable logistic regression analysis, it further confirmed the associations and indicated that children age 7–12 months old (AOR: 0.93, 95%CI: 0.46–2.29, *p* = 0.029), 13–24 months old (AOR: 0.28, 95%CI: 0.14–0.58, *p* = 0.001), and 25–59 months old (AOR: 0.23, 95%CI: 0.12–0.43, *p < 0*.*001)* were less likely to have been exclusively breastfed than children who were 0–6 months. Also, Islamic religion (AOR: 1.86, 95%CI: 1.54–5.42, *p* = 0.033) and Traditionalist (AOR: 3.92, 95%CI: 1.29–5.78, *p* = 0.021) were more likely to have exclusively breastfed their babies compared to Christians (Table 4).

**Table 4:** Associations between background characteristics and EBF.

Variables	EBF		COR (95% CI) p-value		AOR (95% CI) p-value
	Yes	No			
**District**					
Ketu South	68 (45.6)	81 (54.4)	1	0.825	
Hohoe	50 (42.7)	67 (57.3)	0.89 (0.55–1.45)		
Ho West	55 (42.3)	75 (57.7)	0.87 (0.54–1.40)		
**Household size**					
1–5	94 (39.0)	147 (61.0)	1	0.052	
6–10	67 (50.0)	67 (50.0)	1.56 (1.02–2.39)		
11–15	12 (57.1)	9 (42.9)	2.09 (0.85–5.14)		
**Age of mothers (years)**					
<20	22 (51.2)	21 (48.8)	1	0.656	
20–29	77 (43.5)	100 (56.5)	0.74 (0.38–1.43)		
30–39	67 (42.9)	89 (57.1)	0.72 (0.37–1.41)		
≥40	7 (35.0)	13 (65.0)	0.51 (0.17–1.54)		
**Sex of child**					
Male	103 (47.5)	114 (52.5)	1	0.091	
Female	70 (39.1)	109 (60.9)	0.71 (0.48–1.06)		
**Age group of children (months)**					
0–6	58 (59.8)	39 (40.2)	1	**< 0.001**	1
7–12	35 (58.3)	25 (41.7)	0.94 (0.49–1.81)		0.93 (0.46–2.29) **0.029**
13–24	29 (34.5)	55 (65.5)	0.36 (0.19–0.65)		0.28 (0.14–0.58) **0.001**
25–59	51 (32.9)	104 (67.1)	0.33 (0.19–0.56)		0.23 (0.12–0.43) **< 0.001**
**Ethnicity**					
Ewe	162 (44.5)	202 (55.5)	1	0.241	
Akan	1 (14.3)	6 (85.7)	0.21 (0.03–1.74)		
Guan	3 (25.0)	9 (75.0)	0.42 (0.11–1.56)		
Kokomba	4 (66.7)	2 (33.3)	2.49 (0.45–3.79)		
Manprussi	3 (42.9)	4 (57.1)	0.94 (0.21–4.24)		
**Religion**					
Christianity	152 (41.8)	212 (58.2)	1	**0.022**	1
Islam	8 (57.1)	6 (42.9)	1.86 (1.63–5.47)		1.86 (1.54–5.42) **0.033**
Traditionalist	13 (72.2)	5 (27.8)	3.63 (1.31–4.42)		3.92 (1.29–5.78) **0.021**
**Educational Status**					
None	11 (44.0)	14 (56.0)	1	0.153	
Basic	117 (40.9)	169 (59.1)	0.88 (0.39–2.01)		
Secondary	31 (49.2)	32 (50.8)	1.23 (0.49–3.13)		
Tertiary	14 (63.6)	8 (36.4)	2.23 (0.69–4.21)		
**Occupation**					
Artisan	39 (45.9)	46 (54.1)	1	0.193	
Farming	26 (57.8)	19 (42.2)	1.61 (0.79–3.35)		
Government Employee	11 (50.0)	11 (50.0)	1.18 (0.46–3.01)		
Petty trading	70 (38.7)	111 (61.3)	0.74 (0.44–1.25)		
Unemployed	27 (42.9)	36 (57.1)	0.89 (0.46–1.71)		

### 3.5 Associations between healthcare services mothers received towards breastfeeding and EBF

The study showed a significant association between EBF and feeding practice mothers counselled on (*p* = 0.001). Furthermore, when a variable that had an association with EBF was adjusted to eliminate confounding effects, the association was further confirmed and revealed that mothers counselled on complementary feeding (AOR: 0.35, 95%CI: 0.22–3.33, *p* = 0.003) and those who did not remember what they were counselled on (AOR: 0.36, 95%CI: 0.12–1.89, *p* = 0.023) were less likely to exclusively breastfeed their babies than those counselled on mixed feeding. Mothers who were counselled on EBF (AOR: 1.72, 95%CI: 1.26–3.09, *p* = 0.016) were more likely to exclusively breastfeed their babies compared with those counselled on mixed feeding (Table 5).

**Table 5:** Associations between healthcare services mothers received towards breastfeeding and EBF.

Variable	EBF		COR (95% CI) *P*-value		AOR (95% CI) *P*-value
	Yes	No			
**Health facility attended ANC**					
CHPS Centre	10 (47.6)	11 (52.4)	1	0.498	
Health Centre	75 (45.2)	91 (54.8)	0.91 (0.37–2.25)		
District Hospital	66 (43.1)	87 (56.9)	0.84 (0.34–2.08)		
Regional Hospital	7 (28.0)	18 (72.0)	0.43 (0.13–1.45)		
Private Clinic/Hospital	15 (48.4)	16 (51.6)	1.03 (0.34–3.13)		
**Gestational age at first ANC visits (months)**					
1–3	122 (44.0)	155 (56.0)	1	0.712	
4–6	29 (39.7)	44 (60.3)	0.84 (0.49–1.42)		
7–9	4 (57.1)	3 (42.9)	1.69 (0.37–4.71)		
Don’t know	18 (46.2)	21 (53.8)	1.09 (0.56–2.13)		
**Number of ANC visits before delivery**					
Once	6(42.9)	8 (57.1)	1	0.894	
Twice	13 (41.9)	18 (58.1)	0.96 (0.27–3.45)		
Thrice	6 (42.9)	8 (57.1)	1.01 (0.22–4.47)		
Four or more	148 (43.9)	189 (56.1)	1.04 (0.36–3.08)		
**Received advice on baby care at ANC**					
Yes	95 (40.8)	138 (59.2)	1	0.161	
No	78 (47.8)	85 (52.2)	1.33 (0.89–2.99)		
**Feeding practice counselled on**					
Mixed feeding	34 (35.8)	61 (64.2)	1	**0.001**	1
Complement feeding	3 (27.3)	8 (72.7)	0.67 (0.17–2.71)		0.35 (0.22–3.33) **0.003**
EBF	127 (50.6)	124 (49.4)	1.84 (1.13–2.99)		1.72 (1.26–3.09) **0.016**
Don’t remember	9 (23.1)	30 (76.9)	0.54 (0.23–1.97)		0.36 (0.12–1.89) **0.023**
**Number of PNC visits**					
Once	50 (37.9)	82 (62.1)	1		
Twice	18 (34.0)	35 (66.0)	0.84 (0.43–1.65)	0.112	
Thrice	11 (44.0)	14 (56.0)	1.29 (0.54–3.06)		
Four or more	90 (50.3)	89 (49.7)	1.66 (1.05–2.62)		
None	4 (57.1)	3 (42.9)	2.19 (0.47–3.18)		
**Educated on baby care during PNC visit**					
Yes	46 (44.7)	57 (55.3)	1	0.889	
No	127 (43.3)	166 (56.7)	0.95 (0.60–1.49)		

### 3.6 Associations between child feeding and weaning practices and 6 months EBF

The study showed significant associations between EBF and whether mother had ever heard about EBF (*p* = 0.043); babies ever fed from bottles with nipple (*p* = 0.001); prolong breastfeeding (*p* < 0.0001), and age starting complementary feeding (*p* < 0.001). Additionally, when variables that were having associations with EBF from the bivariate analysis were tested for confounding effects using multivariable logistic regression analysis, we found that mothers who had never heard about EBF were less likely to exclusively breastfeed their babies (AOR: 0.46 95%CI: 0.27–4.18, *p* = 0.039) than those who had heard about EBF; babies never fed from the bottle with a nipple were more likely to be breastfed exclusively (AOR: 1.63, 95%CI: 1.94–2.48, *p* = 0.003) than their peers who had ever been fed from bottle with nipple. Mothers who think appropriate age to start complementary feeding is 1–3 months (AOR: 3.40, 95%CI: 1.51–4.83, *p* = 0.021), 4–5 months (AOR: 2.52, 95%CI: 1.47–3.71, *p* = 0.028), 6 months (AOR: 7.43, 95%CI: 3.47–7.55, *p* = 0.001), and mothers who do not know when to start complementary feeding (AOR: 1.62, 95%CI: 1.36–4.76, *p* = 0.008) were more likely to practice EBF than those who think appropriate age to start complementary feeding to be less than a month. Also, children who did not practice prolong breastfeeding were less likely to be associated with EBF (AOR: 0.41, 95%CI: 0.12–0.87, *p* = 0.001) than their peers who were still breastfeeding (i.e. practicing prolong breastfeeding) (Table 6).

**Table 6:** Associations between child feeding and weaning practices and EBF.

Variables	EBF		COR (95% CI) *P*-value		AOR (95% CI) *P*-value
	Yes	No			
**Time breastfeeding started after birth**					
< 1 hour	106 (43.8)	136 (56.2)	1	0.261	
1–5 hours	51 (48.1)	55 (51.9)	1.19 (0.75–1.88)		
6–24 hours	7 (43.7)	9 (56.3)	0.99 (0.36–2.77)		
After a day	9 (28.1)	23 (71.9)	0.50 (0.22–1.13)		
**Mother ever heard about EBF**					
Yes	160 (45.5)	192 (54.5)	1	**0.043**	1
No	13 (29.5)	31 (70.5)	0.51 (0.23–0.98)		0.46 (0.27–4.18) **0.039**
**Times breastfeed/feed infant in a day**					
1–4	20 (33.9)	39 (66.1)	1	0.211	
5–8	30 (39.0)	47 (61.0)	1.24 (0.61–2.53)		
More than 8	46 (46.0)	54 (54.0)	1.66 (0.85–3.24)		
In demand	77 (48.1)	83 (51.9)	1.81 (0.97–3.37)		
**Child ever drunk anything from a bottle with a nipple**					
Yes	66 (34.9)	123 (65.1)	1	**0.001**	1
No	107 (51.7)	100 (48.3)	1.99 (1.33–2.99)		1.63 (1.94–2.48) **0.003**
**Prolonged breastfeeding (n = 241)**					
Yes	98 (58.3)	70 (41.7)	1	**< 0.001**	1
No	24 (32.9)	49 (67.1)	0.35 (0.19–0.62)		0.41 (0.12–0.87) **0.001**
**Appropriate age (months) to start complementary feeding**					
< 1	2 (8.3)	22 (91.7)	1	**< 0.001**	1
1–3	5 (23.8)	16 (76.2)	3.44 (1.59–5.02)		3.40 (1.51–4.83) **0.021**
4–5	14 (18.4)	62 (81.6)	2.48 (1.52–5.81)		2.52 (1.47–3.71) **0.028**
At 6	145 (55.1)	118 (44.9)	3.52 (3.12–6.66)		7.43 (3.47–7.55) **0.001**
Don’t know	7 (58.3)	5 (41.7)	1.54 (1.36–4.71)		1.62 (1.36–4.76) **0.008**
**Duration (months) for breastfeeding a child**					
1–6	39 (46.4)	45 (53.6)	1	0.782	
6–12	11 (35.5)	20 (64.5)	0.64 (0.27–1.49)		
12–18	23 (42.6)	31 (57.4)	0.86 (0.43–1.71)		
18–24	52 (43.0)	69 (57.0)	0.87 (0.49–1.52)		
> 24	41 (47.1)	46 (52.9)	1.03 (0.56–1.88)		
Don’t know	7 (36.8)	12 (63.2)	0.67 (0.24–1.88)		

## 4. Discussion

Preparations towards child care, including breastfeeding and all other feeding practices, are expected to begin during pregnancy. Education towards the achievement of the goal is given at health facilities when mothers visit for ANC services. However, 14.9% of the mothers in this study did not attend the recommended four or more ANC visits for every pregnant woman and did not find any association between ANC visits and EBF. Although ANC visits and services received including childcare and feeding practices are expected to influence child feeding, only 63.4% and 2.8% of the mothers were counselled on EBF and complementary feeding during the ANC visits. A little over quarter (27.8%) of them were counselled on child feeding and care during post-natal care visits in anticipation that there will be an improved child care and feeding practices.

Improvement in child care, including breastfeeding, requires that mothers, especially working mothers get support and encouragement from peers, close relatives, and non-family members. Studies have shown that child care and breastfeeding improves in areas where mothers do receive support, counselling, and regular visits from peers, family, and non-family members [31–33]. Evidence has shown that early initiation of breastfeeding and exclusive breastfeeding in the first month of life can reduce about 23% of neonatal deaths [34]. A causal relationship between early infant feeding practices and infection specific neonatal mortality study showed that new-borns who started breastfeeding within 1 hour of birth were less likely to die of neonatal sepsis than those who did not [35]. Another systematic review study agrees with these finding and stated that infants who initiated breastfeeding 2–23 hours after birth had 33% greater risk of neonatal mortality, and infants who initiated breastfeeding ≥24 hours after birth had about 2.2-fold greater risk of neonatal mortality compare to infants who initiated breastfeeding ≤1 hour after birth [36]. Despite these dangers, only 61.1% of caregivers in the study initiated feeding within an hour of birth and 43.7% of the mothers practised EBF. This is inconsistent with global figures, and what was reported in Ghana’s demographic and health survey and in the Keta-North district in Ghana [1, 11, 12, 37]. A study however has shown that many mothers are facing breastfeeding problems because their traditional source of learning has been reduced as extended families are gradually being replaced by nuclear families, providing little opportunities for caregivers to learning about appropriate breastfeeding [38]. Another challenge has to do with conditions such as formula feeding practices, breast engorgement, sore nipple, nipple trauma, and insufficiency of milk [39]. Hence, to enable a mother to start or continue enjoying the lactation process, prevention and management of these challenges are significant.

Several approaches can be employed to promote exclusive breastfeeding in the Volta region. For instance, an intervention study to explore the influence of peers on exclusive breastfeeding in sub-Saharan Africa concluded that low-intensity individual breastfeeding peer counselling methods could effectively increase the prevalence of EBF in the sub-region [40]. Another intervention study in South Africa indicated that self-efficacy significantly predicted breastfeeding initiation and duration. It concluded that supporting breastfeeding behaviour through programmes that include both individual-level and multi-systems modules aiming the role of healthcare providers, family and community may craft environments that value and support EBF behaviour [41].

The study revealed that 66.4% of the caregivers started complementary feeding at six months. This finding disagrees with the global data, Ghana Demographic and Health Survey, and the Keta-North district study [1, 11, 12, 37]. However, from six months after birth, breastmilk alone is not enough to meet all nutritional needs leading to increased risk of malnutrition among infants. Therefore, adequate and appropriate complementary feeding needs to be started at the sixth month to supplement the breast milk [42]. A study has also shown that the late introduction of complementary feeding can lead to adverse health consequences, including deficiencies of zinc, protein, iron, and the B-vitamins and vitamin D that will lead to growth suppression and cause and rickets [43, 44]. Hence, children need a thoughtful adult who will not only select and offer appropriate foods but also assist and encourage them to consume these foods in sufficient quantities to meet their nutritional needs. This study also indicated that caregivers who started complementary feeding in the sixth month after birth, have a higher chance of practising EBF than caregivers who started complementary feeding before the sixth month, this is not surprising because exclusive breastfeeding should last for at least six months.

Early termination of breastfeeding seems to pose a huge challenge in Africa. This study has shown that 30.3% of the mothers did not practiced prolong breastfeeding, while another study in South Africa reported that 20% of HIV-negative women stopped breastfeeding their children by the third month of birth and called for an urgent need to improve antenatal breastfeeding counselling [45]. Although education and counselling on child feeding and care start from pregnancy and given during ANC visits. The child breastfeeding reported in this study and other parts of Africa could have arisen because caregivers do not attend ANC during pregnancy, and those who do attend do not attend frequently and regularly [32]. For instance, only 69.9% of the mothers attended ANC within the first trimester of gestation. A study however showed that 72.7% of mothers do not even know the right gestation age at which a pregnant woman should start attending antenatal care [46]. This could be due to the availability of health facilities in terms of geographical and financial accessibility as may be corroborated by a study in Nigeria confirming that affordability and proximity of health care facility influences pregnant women’s choice of health institution they attend [47]. Hence, it was not surprising that this study showed that caregivers who terminated breastfeeding before 24 months were less likely to have practised exclusive breastfeeding. Interventions targeted at extending the duration of breastfeeding in populations need to focus on enhancing caregiver’s self-confidence concerning breastfeeding [48].

To enhance breastfeeding practices, caregivers are encouraged not to use pacifiers and bottle-feeding because supplementary feedings, irrespective of the method use (cup or bottle), have a detrimental effect on breastfeeding duration [49]. Notwithstanding, 47.7% of caregivers have ever fed their babies from a bottle with a nipple, and 56.8% were still practising bottle-feeding. Meanwhile, only 4.2% of caregivers are known to follow all the five steps to ensure bottle-feeding is safe [50]. However, bottle-feeding is a known leading cause of diarrhoea [51]. Another study has shown that children need to be protected from cold temperatures because they are at increased risk from cold stress, especially those with low weights [52]. A study reported that infants feel warmer during breastfeeding than bottle-feeding [53].

Health education and health promotion have shown quite substantial effects on behaviour change. However, learning principles including rewards and feedback, that have shown increase effectiveness, are often not adequately applied [54]. This agrees with the present study finding, which revealed that caregivers counselled on child feeding practices was associated with EBF. Another study in North-western Ethiopia, also reported that mothers who received breastfeeding counselling during ANC were more likely to practice EBF than their counterparts [55]. However, a study underscores the need to develop more effective individual interventions than providing broad-based and community-wide health education programmes [56].

Child age was also associated with EBF, with studies in Southern Brazil and South-western Ethiopia which both correlate with the fact that child age is indeed associated with EBF [57, 58]. The associations showed that as a child grows, the rate of exclusive breastfeeding reduces, which may be that caregivers are tempted to give food to children as they crave for food. However, a study has shown that EBF for six months has the potential to reduce child mortality [35]. The study in Brazil [56] agrees with the present study that feeding a child with a pacifier (bottle with nipple) is associated with EBF. A study revealed that children never fed with pacifiers were having a greater chance of being exclusively breastfed, and concluded that children fed with pacifiers are at higher risk of getting an infection than those not fed with pacifiers [51].

Knowledge and awareness of breastfeeding recommendations may greatly influence breastfeeding practices, and this was revealed in this study, which indicated that caregivers who have never heard about EBF are less likely to practice EBF than caregivers who have ever heard. This finding agrees with another study, which indicated that women without the knowledge and awareness of exclusive breastfeeding had a higher risk of ceasing breastfeeding early compared with women who know about breastfeeding [59]. Hence, public health officials need to continue childcare and feeding education unceasingly and involve personalities who matter in maternal and child health issues in the communities in the breastfeeding and child care processes.

Religious involvement can influence several health outcomes as indicated in this study, which revealed that religion was associated with EBF. This further agrees with both the results of the Fragile Families and Child Wellbeing and the United State of America studies [60, 61]. Therefore, understanding religious differences in breastfeeding could enable public health professionals to more effectively handle susceptible populations.

### 4.1 Study limitation

The study could not assess the nutritional status of the children to compare with the feeding and weaning practices to determine the effect of the practices on the nutritional outcomes. We also think a cross-sectional quantitative study may not be enough to explore the actual picture of EBF and the associated factors. Again, recall bias could also affect the study outcome. Notwithstanding, we believe that the limitation cannot invalidate the findings of the study.

## 5. Conclusion

Childcare and breastfeeding education has been going on in the study area and Ghana for decades, yet mothers’ knowledge and practices of breastfeeding revealed in the study are not promising. We, therefore, recommend innovative policies that include extensive public education to improve the breastfeeding and weaning practices of mothers.

## Supporting information

S1 Dataset (XLS)
